# Multi-Material Extrusion-Based 3D Printing of Hybrid Scaffolds for Tissue Engineering Application

**DOI:** 10.3390/gels12020123

**Published:** 2026-01-29

**Authors:** Andrey Abramov, Yan Sulkhanov, Natalia Menshutina

**Affiliations:** Department of Chemical and Pharmaceutical Engineering, Mendeleev University of Chemical Technology of Russia, Miusskaya pl. 9, 125047 Moscow, Russia; yan.sulkhanov.dmi@gmail.com (Y.S.); chemcom@muctr.ru (N.M.)

**Keywords:** extrusion-based 3D printing, direct ink writing (DIW), hydrogel inks, multi-material 3D printing, hydrogel–thermoplastic hybrid scaffolds, tissue engineering, printability and dosing accuracy, process parameters

## Abstract

Additive manufacturing of hydrogel-based scaffolds requires concurrent control of material rheology and extrusion dynamics, especially in multi-material architectures. In this work, we develop a modular multi-material extrusion-based 3D-printing platform that combines a filament-fed extruder for thermoplastic polymers with a piston-driven extruder for viscous gel inks, together with an empirical calibration procedure for gel dosing. The calibration algorithm optimizes the pre-extrusion and retraction displacement (E_Pr_/R) based on stepwise extrusion experiments and reduces the discrepancy between theoretical and measured deposited mass for shear-thinning alginate gels to below the prescribed tolerance. The calibrated system is then used to fabricate two representative hybrid constructs: partially crosslinked sodium alginate scaffolds with an internal hollow channel supported by a removable polycaprolactone framework, and self-supporting structures based on a sodium alginate–chitosan polyelectrolyte complex obtained by sequential co-extrusion. The resulting constructs remain mechanically stable after ionic crosslinking and solvent treatment and can subsequently be converted into highly porous scaffolds by freeze- or supercritical drying. The proposed combination of hardware architecture and extrusion calibration enables reproducible multi-material 3D printing of hydrogel–thermoplastic hybrid scaffolds and can be readily adapted to other gel-based inks for tissue engineering applications.

## 1. Introduction

The implementation of modern technologies and equipment is a key factor in increasing the efficiency of manufacturing processes, improving product quality, and reducing costs. One of the most promising directions in this context is the use of additive manufacturing technologies, or 3D-printing processes [1]. Additive manufacturing encompasses processes based on the layer-by-layer fabrication of parts by depositing material onto a build platform. Three-dimensional printing makes it possible to obtain objects with complex geometries and internal architectures that are difficult or impossible to produce using traditional manufacturing methods such as casting or machining [2].

The most widespread additive manufacturing technologies are those based on extrusion processes [3]. In this case, the formation of a solid structure can occur via various physicochemical transformations such as crystallization, photopolymerization, chemical crosslinking, removal of a volatile solvent, etc. [4,5,6]. At present, two main extrusion-based 3D-printing technologies are distinguished: fused deposition modeling (FDM) and direct ink writing (DIW) [7].

FDM is used to fabricate parts from thermoplastic polymers—high-molecular-weight compounds that soften upon heating and transition to a viscous-flow state, while recovering their initial characteristics upon cooling [8]. In turn, when fabricating parts from thermolabile materials, viscous gels, or dispersed systems, direct ink writing is employed [9,10]. The key distinction of this approach is the absence of any need to heat the polymer prior to its deposition onto the build platform. In DIW, the geometry of the final part is formed due to the specific rheological properties of the used materials, namely shear-thinning behavior, thixotropy, and the presence of a yield stress [11,12,13,14,15]. Extrusion-printable hydrogels have also been developed for controlled ocular drug delivery, demonstrating the potential of DIW-based systems for biomedical applications [16].

In recent years, extrusion-based additive manufacturing has attracted growing attention from researchers for the fabrication of parts with functional properties by combining several materials within a single technological process. One way to achieve this is to employ two independent extruder modules that process materials with different physicochemical characteristics within one printing run [17]. The multi-material 3D-printing process is based on the sequential deposition of materials, which allows the part to be divided into functional zones with tailored properties. This approach significantly broadens the application area of the fabricated parts by improving their biological performance, electrical conductivity, and mechanical characteristics [18,19]. Recent comprehensive reviews summarize advances in 3D printing of polymer composites and multi-material systems, with particular emphasis on structure–property relationships and process optimization [20].

For example, in [21] scaffolds for tissue engineering applications were obtained using viscous inks based on sodium alginate in combination with a thermoplastic polymer, polycaprolactone, which was employed to enhance the mechanical properties of the final construct. In addition, the material formulations for multi-extrusion 3D printing can be selected such that a chemical reaction occurs between them, which can be used to fix the structure and eliminate the need for additional post-processing steps [22].

Sodium alginate and chitosan are among the most widely studied biopolymers for biomedical applications. Alginate-based hydrogels and composites have been extensively investigated as scaffolds for tissue engineering and regenerative medicine, where they can provide a hydrated, ECM-mimicking environment that supports cell adhesion, proliferation, and differentiation [23,24]. Chitosan, owing to its cationic nature and structural similarity to glycosaminoglycans, has been shown to promote cell attachment and to endow materials with intrinsic antibacterial activity [25,26]. Scaffolds based on alginate–chitosan polyelectrolyte complexes, including freeze-dried and 3D-structured constructs, have demonstrated non-cytotoxic behavior and the ability to support cell growth in various in vitro and in vivo models, highlighting their potential as bioactive matrices in tissue engineering [27,28]. These studies provide a materials platform of biocompatible and biodegradable polymers that is well suited for integration with advanced extrusion-based 3D-printing strategies.

A key factor determining the quality of printed parts is precise control over material dosing during 3D printing. However, the extrusion of viscous gel materials through small-diameter nozzles is associated with technological challenges caused by the inertia of the feeding mechanism and the viscoelastic behavior of the materials [29,30]. These phenomena and processes may lead to insufficient material extrusion during part formation, resulting in thinning and breakage of deposited filaments, as well as spontaneous oozing of the material after extrusion is stopped. To improve print quality, various approaches are used to select process parameters, including methods based on machine learning [31], mathematical modeling [32,33], and the implementation of additional control loops [30]. Of particular interest are calibration methods based on the sequential variation of the system control parameters. Their use makes it possible to avoid complex analytical studies, since the required operating mode of the equipment is determined experimentally by comparing the actual and target dosed mass, which greatly simplifies process setup.

In this work, we present equipment for implementing additive manufacturing processes, namely a multi-extrusion 3D-printing process using thermoplastics and biopolymer-based gel materials. In addition, we propose a calibration procedure for the gel extruder based on optimizing the pre-extrusion and retraction length parameters. To assess the applicability of the proposed calibration method and the technical capabilities of the developed setup, a 3D-printing study was carried out in which complex-shaped parts were fabricated, namely: (1) a part with an internal hollow channel based on sodium alginate and the thermoplastic polymer polycaprolactone; and (2) a part based on a polyelectrolyte complex formed by sequential extrusion of sodium alginate– and chitosan-based gel materials. Thus, the present manuscript is focused on the process engineering aspects of multimaterial extrusion-based 3D printing with biocompatible inks, while the detailed biological evaluation of cell behavior on the printed constructs is reserved for future studies.

## 2. Results and Discussion

**Figure 1:**
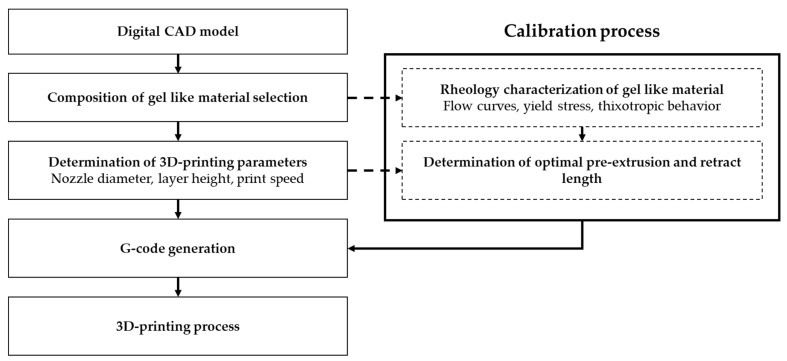
Block diagram of the manufacturing process for parts using gel-based materials and 3D printing.

The fabrication of parts using multi-material 3D-printing technology is a process of sequentially building the geometry by depositing layers of materials with different compositions. The 3D-printing process involving gel-based materials can be represented in the form of a block diagram that describes the sequence of the required operations (Figure 1).

The 3D-printing process begins with the design of a digital model with complex geometry using computer-aided design (CAD) systems. Next, depending on the required macro- and mesoporous structure of the final part, suitable materials are selected for the printing process. At the following stage, the process parameters are defined, namely the printing head travel speed, the nozzle orifice diameter, and the layer height. These parameters are specified by the user and depend on the desired properties of the final product. It should be noted that the flow rate of the gel materials during extrusion, which is governed by the rheological properties of the material and the process conditions, has a significant influence on the printing process. Based on the selected process parameters, a sequence of G-code commands is generated and sent to the 3D printer control board, after which the part fabrication process is carried out.

### 2.1. Calibration Process of Extrusion Gel-like Materials

**Figure 2:**
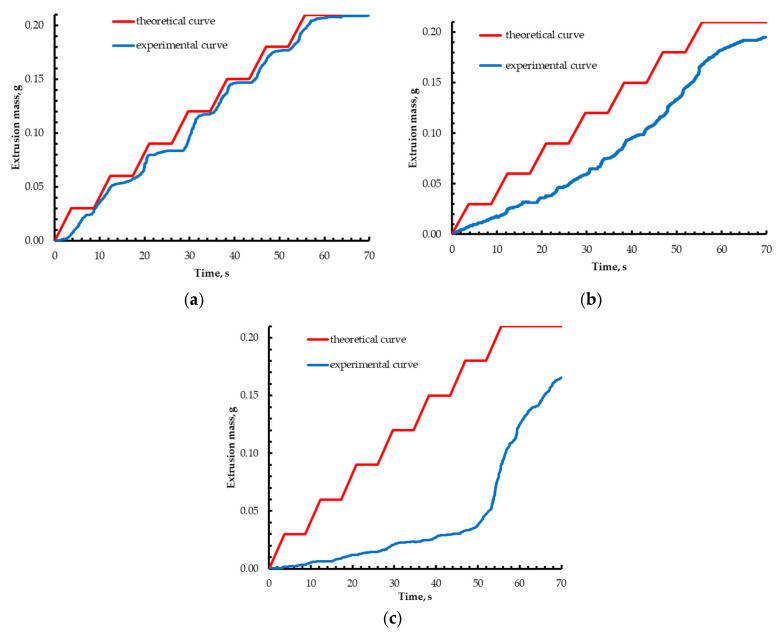
Experimental and theoretical extrusion curves for pure water (**a**), partially crosslinked sodium alginate with a concentration of 0.05 wt% (**b**), and partially crosslinked sodium alginate with a concentration of 0.20 wt% (**c**).

Stepwise extrusion experiments (Section 4.4) were carried out for partly crosslinked alginate based on 2 wt% sodium alginate with added CaCl_2_, as well as for a reference sample of pure water. The experiment consisted of 7 extrusion cycles, each delivering 0.3 g of material at a constant plunger speed of 0.0035 mm/s, with a 5 s pause between cycles. This setup makes it possible to assess the accumulation of mass deviation when using gel materials with different rheological properties (Figure 2).

**Figure 3:**
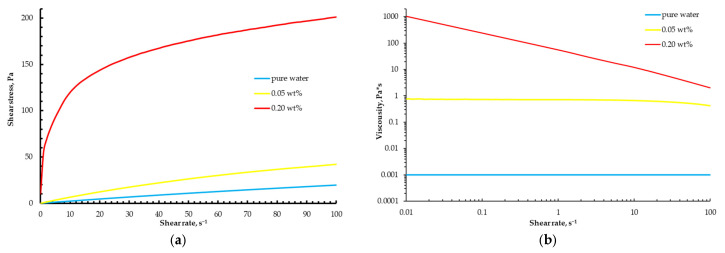
Viscosity (**a**) and flow (**b**) curves of the materials used.

The largest deviation from the theoretical curve is observed for the material with the addition of 0.2 wt% calcium chloride (Figure 2c). The shape of the obtained curves and the magnitude of their deviation from the theoretical dependence reflect the differences in the rheological properties of the materials (Figure 3).

An increase in calcium chloride concentration markedly raises the solution viscosity due to the formation of a stable polymer gel network. In addition, the flow behavior of the “ink” changes, and an increase in the degree of shear-thinning is observed, which is reflected in the slope of the flow curves. However, establishing a direct quantitative relationship between the rheological parameters of the material and the magnitude of the extrusion deviation is a complex analytical task, owing to the multitude of concurrent processes during extrusion—local pressure losses, viscosity reduction in the ink followed by relaxation, and the inertia of the feeding mechanism. To ensure accurate dosing of gel materials, an algorithm for extruder calibration was proposed.

**Figure 4:**
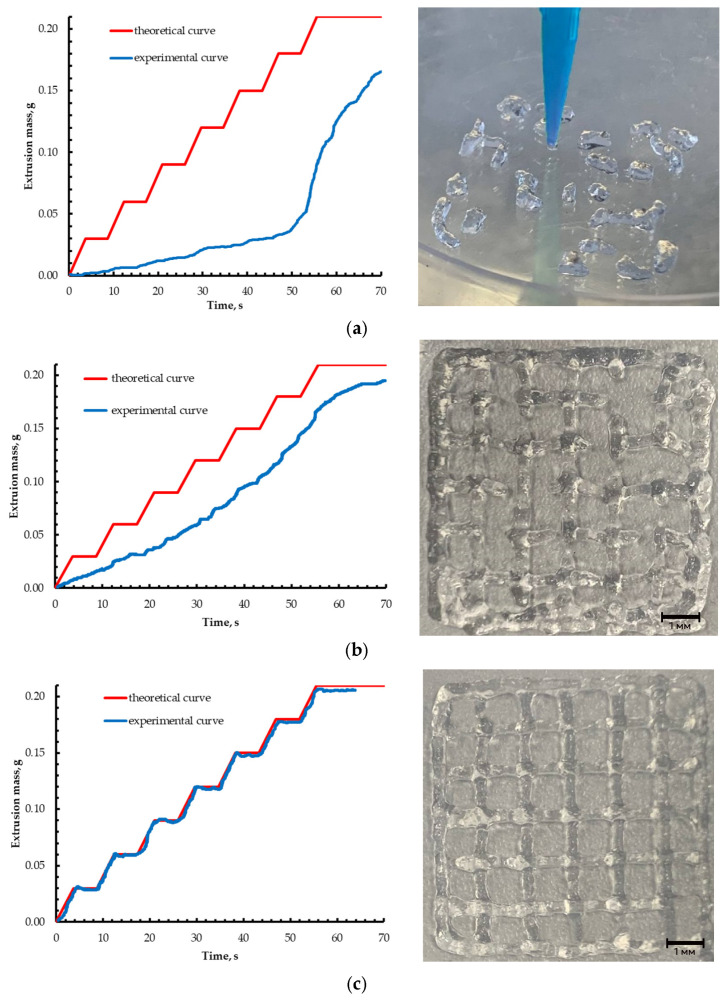
Influence of the offset parameter E_Pr/R_ (mm) on the extrusion of gel materials and the corresponding 3D-printed structures: (**a**) E_Pr/R_ = 0; (**b**) E_Pr/R_ = 0.051; (**c**) E_Pr/R_ = 0.115.

Based on the preliminary studies, the printing parameters were optimized for the gel formulation exhibiting the highest degree of shear-thinning behavior, namely 2 wt% sodium alginate and 0.2 wt% CaCl_2_. The determination of the additional displacement length E_Pr/R_ was carried out with a step ΔE_Pr/R_ of 0.001 mm and a required accuracy ε of 0.01. For the selected formulation, experimental curves of the deposited mass as a function of time were obtained. In addition, to assess the applicability of the proposed approach, a direct ink writing process was performed to fabricate a lattice structure with dimensions of 10 mm × 10 mm × 0.5 mm and a spacing of 1.2 mm between adjacent filaments at different displacement values E_Pr/R_ (Figure 4).

When analyzing the performance of the algorithm and varying the controlled parameter, the experimental values approached the prescribed mass setpoints, and the target accuracy was achieved at a displacement value of 0.115 mm. In this case, the mass deviation, calculated as the average over all extrusion cycles, was 0.006 g. Similar convergence behavior was observed for the other ink compositions tested: in all cases the iterative procedure reached the prescribed tolerance ε within a few iterations, and the mass deviation at the end of each cycle remained within the predefined bounds. The calibration algorithm thus effectively compensates for the combined effects of ink viscoelasticity and feeder inertia without requiring an explicit constitutive model. This empirical, yet systematic approach is readily transferable to other gel-based inks and printing setups, provided that the stepwise extrusion experiment can be reproduced under the same geometric conditions.

The printed samples, in turn, demonstrate that pre-extrusion and retraction have a pronounced effect on part formation and make it possible to improve the quality of 3D printing. At E_Pr/R_ = 0 (Figure 4a), virtually no continuous extrusion was observed, and only isolated segments of material were deposited on the build platform, which did not form an integral lattice. When the displacement was increased to E_Pr/R_ = 0.051 mm (Figure 4b), the extrusion process became more stable; however, gaps between the filaments remained in the resulting structure, indicating an insufficient volume of extruded gel material. At E_Pr/R_ = 0.115 mm (Figure 4c), the lattice structure was formed uniformly, with well-defined filaments of uniform thickness and an adequate material supply throughout the entire process. These results indicate that the proposed solution can significantly improve the quality of the printed parts and stabilize the extrusion of gel materials during 3D printing process.

### 2.2. Multimaterial 3D Printing Process

The proposed extrusion calibration approach was used for the experimental investigation of the formation of complex-shaped parts by multi-material extrusion-based 3D printing. Within this study, two fundamentally different part configurations were fabricated:

- Parts with internal hollow channels formed using gel materials based on partially crosslinked sodium alginate, with a supporting framework made of a thermoplastic polymer.
- Parts based on a polyelectrolyte complex (PEC) of the sodium alginate–chitosan system, obtained by sequential extrusion of the corresponding polymer solutions.

#### 2.2.1. Object with Hollow Channels

**Figure 5:**
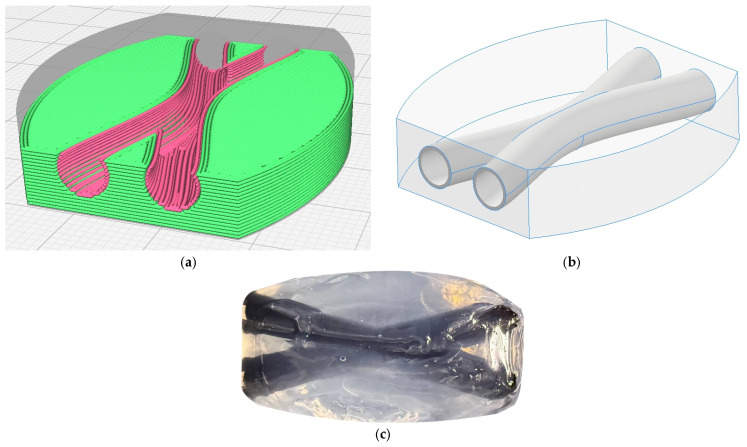
Visualization of the layer structure used in the process (**a**) and the part geometry designed for the implementation of the multi-extrusion 3D-printing process (**b**), part based on partially crosslinked sodium alginate with an internal hollow channel after completion of the gelation process (**c**).

In the implementation of the multi-material 3D-printing process aimed at fabricating a part with an internal hollow channel, extrusion modules for both the thermoplastic polymer and the gel materials were employed. Partially crosslinked sodium alginate was used as the primary material of the construct. It should be noted that, despite its suitable rheological properties, the formation of channels using only the gel material is not feasible. To prevent channel collapse during the fabrication of overhanging structures, the thermoplastic polymer polycaprolactone was used to form the channel walls. The proposed three-dimensional part geometry was imported into dedicated slicing software (UltiMaker Cura 5.11) to generate the corresponding set of control commands for the 3D printer (Figure 5a,b).

Regardless of the physicochemical properties of the materials, the printing process was carried out at a printing speed of 10 mm/s and a layer height of 0.4 mm. To form the main framework of the part based on partially crosslinked sodium alginate, the plunger speed of the gel extruder was set to 0.035 mm/s, with a nozzle orifice diameter of 0.41 mm and a calibration value E_Pr/R_ of 0.115. The walls of the hollow channel made of polycaprolactone were printed at an extruder temperature of 80 °C and a nozzle diameter of 0.4 mm.

The formation of a stable three-dimensional structure after printing was completed by inducing gelation of sodium alginate in a 1 wt% calcium chloride crosslinking solution for 1 h (Figure 5c).

To remove the rigid polycaprolactone framework forming the channel walls, dichloromethane was flushed through the internal channel from a syringe until no visible PCL residues remained. During this procedure, fresh solvent was supplied continuously to ensure complete dissolution of the PCL.

#### 2.2.2. Object Based on a Polyelectrolyte Complex

In the fabrication of the part based on a polyelectrolyte complex, two gel-extrusion modules were used. The stability of the construct during printing is ensured by the formation of a hybrid structure due to the chemical reaction between the polyanion sodium alginate and the polycation chitosan, resulting in the corresponding polyelectrolyte complex.

**Figure 6:**
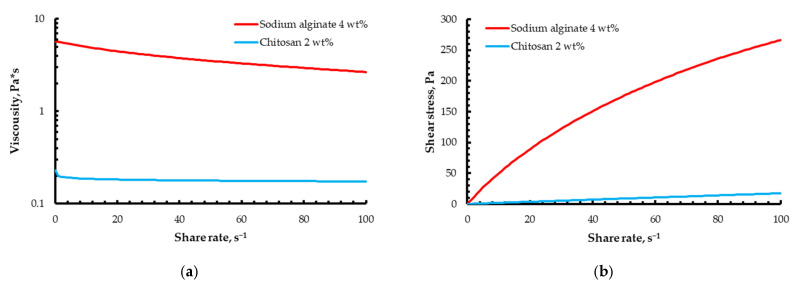
Viscosity (**a**) and flow (**b**) curves of the 4 wt% of sodium alginate and 2 wt% of chitosan.

The rheological behavior of the 4 wt% sodium alginate and 2 wt% chitosan inks used for the polyelectrolyte complex construct is summarized in Figure 6.

Both formulations exhibit pronounced shear-thinning behavior, with viscosities in the range typically reported for printable hydrogel-based inks, which facilitates co-extrusion and shape retention during direct ink writing.

**Figure 7:**
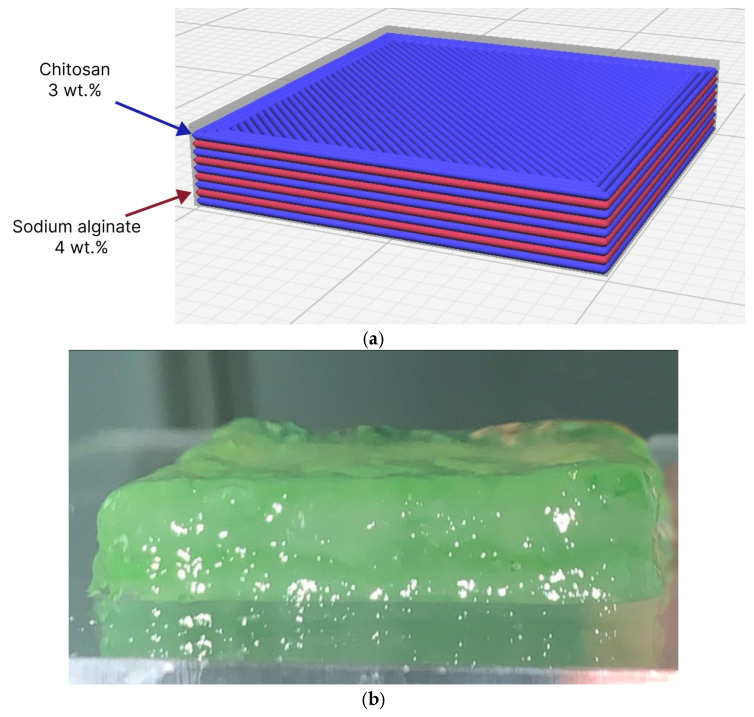
Visualization of the material layers in the part based on a sodium alginate–chitosan polyelectrolyte complex (**a**), Part based on the polyelectrolyte complex after completion of the 3D-printing process (**b**).

For the printing process, a geometry with characteristic dimensions of 20 mm × 20 mm × 5 mm (L × W × H) was designed in a computer-aided design system. The printing procedure involved the sequential deposition of sodium alginate and chitosan layers with equal layer thickness (Figure 7).

Experimental studies of the printing process were carried out under the following conditions: layer height 0.4 mm, nozzle orifice diameter 1.67 mm, and printing speed 10 mm/s. The pre-extrusion and retraction length E_Pr/R_, determined in accordance with the proposed calibration procedure, was 0.085 for sodium alginate and 0.009 for chitosan.

Upon completion of the printing process, a stable construct was obtained that required no additional post-processing, which indicates that the formation of the polyelectrolyte complex had been completed (Figure 7b).

To generate a porous structure, the obtained part can be subjected to supercritical or freeze-drying processes, including the necessary preparatory steps.

From a tissue-engineering perspective, the two demonstrator architectures target complementary use cases. The partially crosslinked sodium alginate constructs with an internal hollow channel and a removable polycaprolactone framework are promising as perfusable scaffolds for vascularized tissues or for dynamic perfusion culture in bioreactors, where the mechanically robust thermoplastic shell maintains the lumen during handling and perfusion. In contrast, the sodium alginate–chitosan polyelectrolyte complex provides an all-hydrogel environment with tunable charge density and stiffness, which is attractive for soft-tissue regeneration (e.g., cartilage-like or intervertebral disk-like constructs). Although no biological experiments are reported in this work, the choice of sodium alginate and sodium alginate–chitosan polyelectrolyte complexes is supported by previous studies showing that aerogels and scaffolds based on these polymers can be non-cytotoxic, promote cell attachment, and provide antimicrobial effects, making them attractive candidates for tissue-engineering applications [23,24,25,26,27,28,34,35,36,37]. The constructs obtained using the proposed multimaterial 3D printing routes can additionally be subjected to freeze-drying or supercritical-drying processes to generate highly porous structures suitable for use as tissue-engineering scaffolds.

## 3. Conclusions

Additive manufacturing is a versatile tool for solving a wide range of problems, including in medicine, pharmaceutics, and chemical engineering. A promising and relevant direction is the use of additive manufacturing technologies for the design and development of new materials with tailored functional properties, which can significantly broaden their application areas. The present study investigates multi-material 3D printing processes aimed at creating composite or hybrid structures using materials with different physicochemical properties within a single technological process. To implement this approach, an additive manufacturing setup was designed and constructed. It provides a large build volume for the fabrication of final parts and allows processing both thermoplastic materials (with melting temperatures up to 280 °C) and viscous inks of various compositions with viscosities up to 2500 Pa·s.

To determine the flow rate of gel materials during the printing process, a calibration method was developed. The applicability of the proposed method was demonstrated in experimental studies and in the optimization of printing parameters for parts fabricated using gel materials based on partially crosslinked sodium alginate. The developed approach was subsequently employed for the implementation of multi-material 3D printing.

Using the setup architecture and extruder calibration method presented in this work, a multi-material 3D printing process was implemented. The possibility of fabricating a part with an internal hollow channel based on partially crosslinked sodium alginate and a thermoplastic polymer (polycaprolactone) is demonstrated. In addition, to illustrate the possibility of combining viscous inks within a single process, the fabrication of parts based on a polyelectrolyte complex is demonstrated. The resulting complex-shaped parts can be subjected to supercritical or freeze-drying to form a highly porous internal structure, which may promote cell proliferation and growth.

Overall, the present work should therefore be viewed as an enabling technological platform for multimaterial extrusion-based 3D printing with biocompatible inks, while future studies will focus on quantifying how the geometry and structural characteristics of the printed constructs influence the adhesion, proliferation and function of cells seeded on these architectures.

## 4. Materials and Methods

### 4.1. Materials

Sodium alginate (CAS Number: 9005-38-3, Sigma-Aldrich, St. Louis, MO, USA) and acid-soluble chitosan with a molecular weight of 111 kDa (Sigma-Aldrich, St. Louis, MO, USA) were used as precursors for the gel materials. The fused deposition process was carried out using a commercially available thermoplastic polycaprolactone (eSUN, Shenzhen, China) supplied as a filament. Other materials, including distilled water and calcium chloride, were purchased from RusChem (Moscow, Russia).

### 4.2. Method of Ink Preparation and Rheology Measurement

Gel materials based on partially crosslinked sodium alginate for extrusion experiments and 3D-printing were prepared using a rotor–stator homogenizer (T 25 digital ULTRA TURRAX, IKA, Dortmund, Germany). A predetermined amount of calcium chloride was dissolved in water to obtain a concentration of 0.2 wt% and stirred until the salt was completely dissolved. Sodium alginate was then added to the resulting solution to reach a concentration of 2 wt%, while mixing at 13,000 rpm for 7 min.

For the multi-material printing experiments, sodium alginate solution with a concentration of 4 wt% (viscosity 5.7 Pa·s) and chitosan solution with a concentration of 2 wt% (viscosity 0.2 Pa·s) were selected. The chitosan-based formulation was prepared by dissolving 2 wt% chitosan in 0.1 M acetic acid. Similarly, the 4 wt% sodium alginate solution was obtained by dissolving sodium alginate in distilled water.

Before starting the 3D-printing process, air bubbles formed during gel preparation were removed from all materials using a centrifuge (C 2201 S-20-15, ELMI, Riga, Latvia) operated at 2500 rpm for 5 min.

The viscosity of the polymer solutions used was measured with a rotational rheometer (SmartPave 102e, Anton Paar, Zurich, Switzerland) equipped with a cone–plate geometry (diameter 50 mm), at a constant temperature of 20 °C and a shear rate of 0.1 s^−1^.

### 4.3. Installation for 3D Printing Process

**Figure 8:**
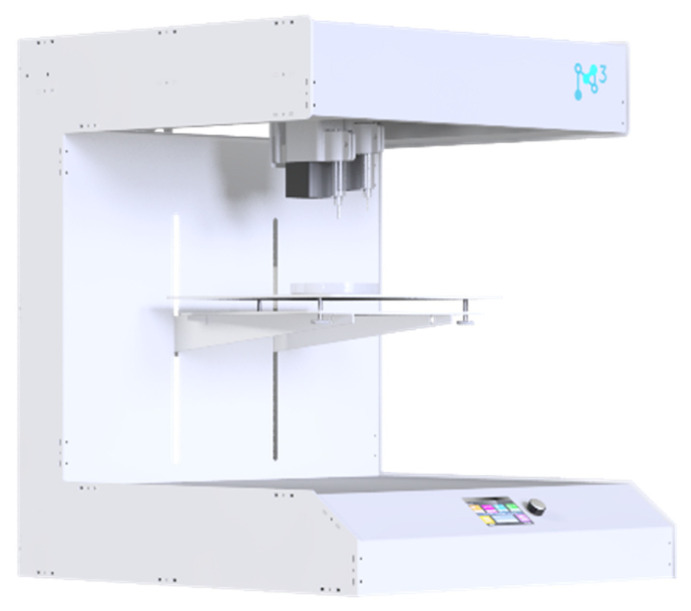
Modular setup for implementing multi-material 3D printing.

In this study, a custom-built setup was used to implement the multi-material 3D-printing process (Figure 8).

**Figure 9:**
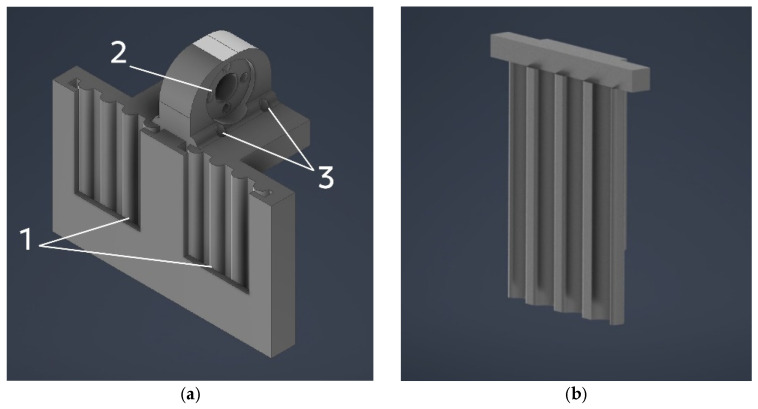
Ensuring the modularity of the additive manufacturing equipment: (**a**) design of the 3D-printer carriage: 1—slot for mounting the extruder; 2—hole for the trapezoidal nut; 3—holes for mounting on the linear guide; (**b**) mating slot component of the extruder module.

The multi-material 3D-printing setup provides a build volume of 300 mm × 300 mm × 350 mm (L × W × H). A distinctive feature of the design is its modular mounting system, which enables rapid reconfiguration of the 3D printer to address different manufacturing tasks. To achieve this, a carriage design was proposed, on which the extruders are mounted using a slot-type connection (Figure 9).

The required module is installed on the moving carriage (Figure 9a) using a specially designed slot connection (1). For the positioning mechanism, the carriage is attached to the rail guide (2) and the trapezoidal nut (3) by bolted joints. In addition, a matching counterpart of the slot connection was designed (Figure 9b), which is subsequently used in the extruder modules.

#### 4.3.1. Description of Extruder for Thermoplastic Polymer

**Figure 10:**
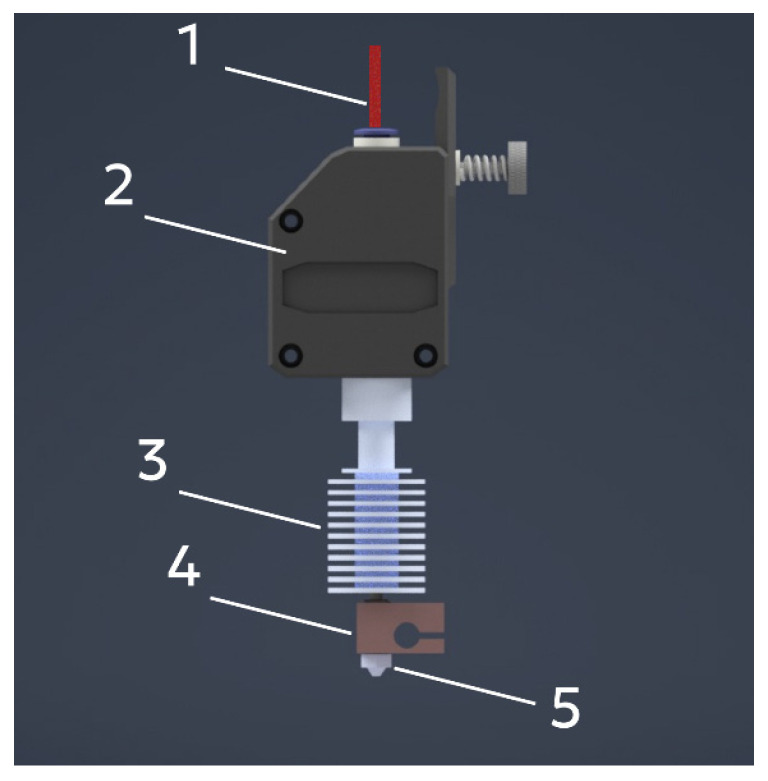
Extruder for thermoplastic polymer: 1—polymer filament; 2—feeding mechanism; 3—heat sink; 4—heating block; 5—nozzle.

For 3D printing with thermoplastic polymers, a filament-fed extruder was used due to its simple design and the broad commercial availability of filament materials (Figure 10).

**Figure 11:**
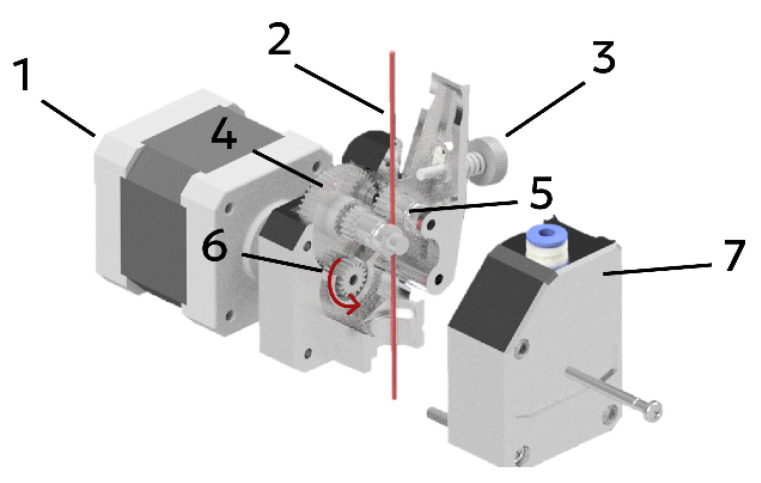
Feeding mechanism in the thermoplastic polymer filament extruder: 1—stepper motor; 2—filament; 3—adjustment screw; 4—idler gear; 5—pressure roller; 6—drive gear; 7—cover.

Initially, the material (1) in the form of a filament enters the feeding mechanism (2) and gradually moves along the extruder until it reaches the heating block (4). In this zone, the polymer melts and is delivered onto the build platform through the nozzle (5). Heating is provided by an integrated cartridge heater and a thermistor-based temperature sensor. In addition, the design is equipped with a heat sink (4) to remove excess heat outside the heating zone. In the presented configuration, material feeding is achieved by the rotation of gears that grip the filament between them (Figure 11).

During material loading, the polymer filament (2) is clamped between the pressure roller (5) and the idler gear (4) using the adjustment screw (3). Feeding of the polymer filament occurs when the drive gear (6), which is mounted on the stepper motor (1), rotates. The assembly is enclosed in a housing; after the main components are installed, the cover (7) is mounted.

#### 4.3.2. Description of Extruder for Gel-like Materials

**Figure 12:**
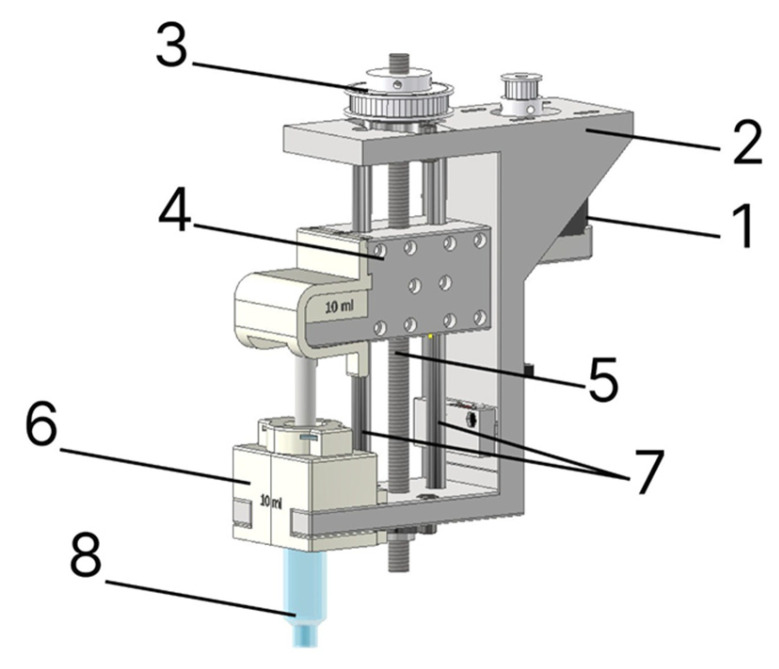
Design of the extruder for 3D printing with gel materials: (1) stepper motor; (2) module housing; (3) belt drive; (4) plunger element; (5) lead screw; (6) linear guides; (7) holder for the material reservoir; (8) material reservoir.

For the direct ink writing process, a piston-driven extruder configuration was developed (Figure 12).

In the upper part of the module housing (2), mounting holes are provided for attaching the stepper motor (1) using four M3 × 25 bolts. To transmit torque from the stepper motor to the trapezoidal screw (5), a belt drive with a 1:3 ratio (3) is used, consisting of two pulleys with diameters of 12 and 36 mm, respectively. The rotational motion is then transferred to the lead screw (5), which drives the plunger element (4). As it moves downward along the linear guides (6), material is dispensed from the reservoir (8), which is fixed to the module housing (2) by a dedicated holder (7).

**Figure 13:**
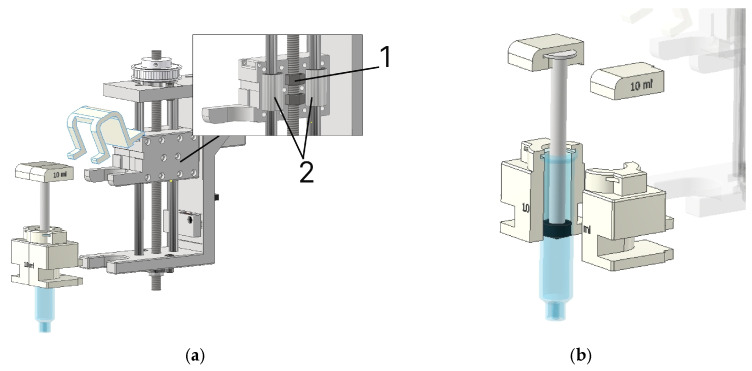
Design of the plunger assembly: (**a**) carriage: 1—nut; 2—rolling bearing; (**b**) syringe adapter.

The plunger element is a composite assembly consisting of two parts: a carriage and an adapter for a 10 mL syringe (Figure 13).

The carriage of the plunger assembly is a composite structure consisting of two parts fastened together with thirteen M3 × 18 screws (Figure 13a). The internal space of the assembly is designed to accommodate two M8 nuts (1), which provide motion of the carriage along the *Z*-axis, as well as two rolling bearings (2) for the linear guides. To secure the reservoir for the gel materials, a dedicated syringe adapter was designed, which is mounted on the carriage (Figure 13b). Owing to the absence of threaded connections, the proposed design enables rapid replacement of the material and allows syringes of different volumes to be used for loading.

### 4.4. Method of Extrusion Calibration

Calibration of the equipment for direct ink writing is carried out on the basis of experimental studies of the extrusion process. During calibration, a stepwise material feed is considered, in which extrusion occurs periodically. This method is close to real printing conditions: each step corresponds to the deposition of an individual filament and makes it possible to quantitatively assess how the mass of extruded material changes under repeated on/off switching of the feed. The dosing accuracy is evaluated by comparing the actual mass of the deposited material with the prescribed theoretical value.

The extruder with the mounted syringe was fixed above an analytical balance (Ohaus PR 200, Ohaus, Parsippany, NJ, USA). A beaker filled with water was placed on the balance, and the extruder nozzle was partially immersed in the water to reduce surface tension forces acting on the gel materials during extrusion. In this configuration, the syringe, tubing, and nozzle geometry were kept constant for all experiments, and the nozzle was partially immersed in water to minimize additional capillary forces. As a result, variations in the experimental mass curve M_exp_(t) primarily reflect the combined influence of the ink rheology (viscosity reduction and relaxation) and the inertia and compliance of the feeding mechanism. In the present work these effects were not decoupled explicitly; instead, their net contribution is accounted for through the empirically adjusted displacement parameter E_Pr/R_.

**Figure 14:**
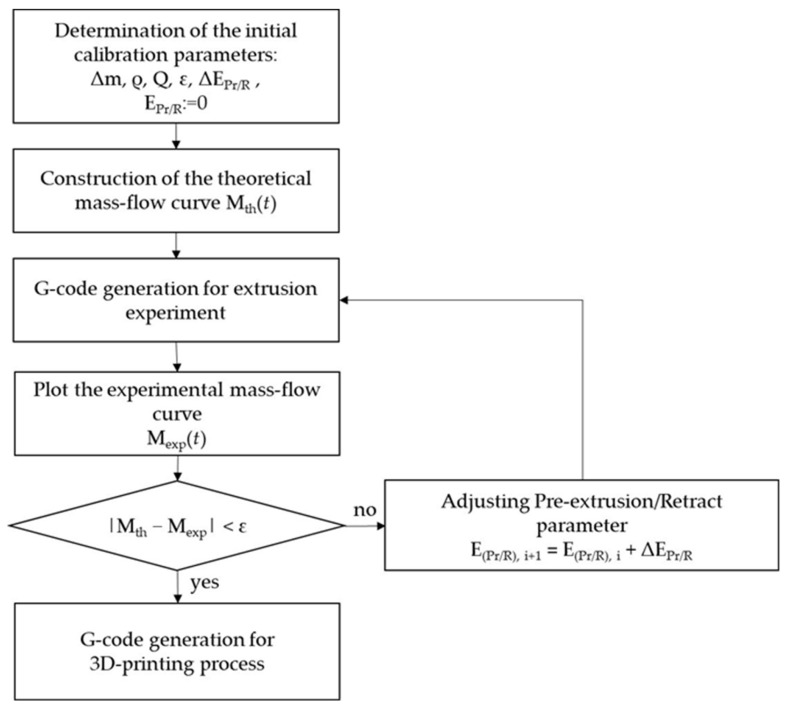
Algorithm for calibrating additive manufacturing equipment for printing with gel materials.

To compensate for the discrepancy between the experimental and theoretical extrusion curves, an algorithm was developed to optimize the flow rate of gel materials as a function of their rheological properties. This algorithm is based on introducing an additional piston displacement parameter, E_Pr/R_, during pre-extrusion and retraction, defined as the distance traveled by the piston (in mm). Pre-extrusion is understood as preliminary material extrusion required to reduce the viscosity of the material before the start of printing. Retraction is the reverse piston motion aimed at reducing the pressure inside the extruder and preventing material oozing after the end of extrusion. The optimal values of the displacement parameter were determined according to the developed procedure (Figure 14).

The algorithm is implemented using an iterative approach. At each step, a control program is generated with a specified value of the E_Pr/R_ parameter. Based on the experimental results, the deviation between the theoretically calculated and experimentally measured material mass is evaluated, after which the value of EPr/R is adjusted until the desired dosing accuracy is achieved. A detailed description of the algorithm steps is given below.

In the first step, the input data required for the optimization algorithm are defined. The target mass Δm of the material with density ρ is specified, which is to be dosed at a given flow rate Q. The allowable error ε is set, defining the maximum permissible deviation between the theoretical and experimentally measured mass. In addition, the step parameters of the algorithm are assigned: the increment ΔE_Pr/R_, which determines the change in the displacement value between successive iterations, as well as the initial value used as the first approximation. In this way, a set of initial parameters is formed that define the starting conditions and the accuracy of the algorithm.

At the next stage, the theoretical dependence of the dispensed material mass on time, Mth(t), is calculated for the selected material and printing parameters. The theoretical curve can be expressed as follows (Equitation (1)):

(1) $$\left\{\begin{array}{c} M_{th}\left(t\right)=0,t<t_{0}\\ M_{th}\left(t\right)=\rho Qt,t_{0}<t\leq t_{1}\\ M_{th}\left(t\right)=\Delta m,t>t_{1} \end{array}\right.$$

where t_0_ is the start time of extrusion and t_1_ is the end time of extrusion.

The resulting theoretical curve Mth(t) describes the amount of material that should be extruded at each moment in time. This curve is used as a reference for subsequent comparison with the experimental data.

Using the parameters defined above and the theoretical model, a set of G-code control commands is generated for the extrusion of the gel materials. During execution of the program, the experimental dependence of the extruded material mass on time M_exp_(t) is recorded. The theoretical curve Mth(t) is then compared with the experimental dependence M_exp_(t). As the optimality criterion, the absolute mass deviation |Mth(t) − M_exp_(t)| is used, which is evaluated at the end of each extrusion cycle.

If the condition |Mth(t) − M_exp_(t)|< ε is not satisfied, the algorithm proceeds to the stage of adjusting the displacement parameter. The value of E_Pr/R_ is updated according to the following expression:

(2) $$E_{\left(\mathrm{Pr}/R\right),i+1}=E_{E_{\left(\mathrm{Pr}/R\right),i}}+E_{\mathrm{Pr}/R}$$

The updated value of E_Pr/R_ is then substituted into the G-code generation block, a new set of control commands is formed, and the experiment is repeated. If the calculated deviation does not exceed the specified tolerance ε, the selected value of E_Pr/R_ is considered to provide sufficient dosing accuracy for the material under study. In this case, the algorithm terminates the iterative process, and the obtained value of the coordinate is taken as optimal and is subsequently used for the 3D-printing process.

In summary, the proposed calibration algorithm uses as input the target mass per extrusion cycle Δm, the density ρ of the ink, the prescribed average flow rate Q, the tolerance ε for the acceptable mass deviation, the increment Δ(E_Pr/R_) between successive iterations, and an initial estimate of E_Pr/R_. The main assumption is that, for a fixed printer configuration and a given ink composition, the difference between the theoretical and experimental mass curves is predominantly governed by the additional piston displacement during the pre-extrusion and retraction phases. The combined effects of local pressure losses, viscosity reduction and relaxation of the gel, as well as the inertia and compliance of the feeding mechanism, are therefore lumped into the empirical parameter E_Pr/R_. The algorithm was validated by repeating the stepwise extrusion experiment until convergence within ε was achieved and by printing representative lattice structures, for which the measured mass of the final parts agreed with the theoretical value within the experimental uncertainty of weighing.

## Data Availability

The original contributions presented in this study are included in the article. Further inquiries can be directed to the corresponding author.
